# Supplementary material for: L-Arginine Ameliorates Defective Autophagy in GM2 Gangliosidoses by mTOR Modulation
Source: Cells. 2021 Nov 11;10(11):3122. doi: 10.3390/cells10113122 (PMC8619250; doi:10.3390/cells10113122)
Supplement: Supplementary file 1 [file cells-10-03122-s001.zip › cells-1441099-supplementary.pdf]

Supplementary data

L-arginine ameliorates defective autophagy in GM2 gangliosidoses by mTOR modulation

Beatriz Castejón-Vega[1], Débora Lendines-Cordero[1], Alejandro Rubio[2], Antonio J. Pérez-Pulido[2], José L. Quiles[3], Jon D. Lane[4], Beatriz Fernández-Domínguez[5], María Begoña Cachón-González[6], Carmen Martín-Ruiz[7], Alberto Sanz[8], Timothy M. Cox[6], Elísabet Alcocer-Gómez[9], Mario D. Cordero[10]

[1] Research Laboratory, Oral Medicine Department, University of Sevilla, Sevilla, Spain

[2] Centro Andaluz de Biologia del Desarrollo (CABD, UPO-CSIC-JA). Facultad de Ciencias Experimentales (Área de Genética), Universidad Pablo de Olavide, 41013, Sevilla, Spain.

[3] Department of Physiology, Institute of Nutrition and Food Technology "José Mataix Verdú", Biomedical Research Center, University of Granada, Granada, Spain.

[4] Cell Biology Laboratories, School of Biochemistry, University of Bristol, Bristol, UK

[5]Acción y Cura para Tay-Sachs (ACTAYS), Madrid, Spain

[6] Department of Medicine, University of Cambridge, Cambridge, UK.

[7] Biosciences Institute, Newcastle University, Newcastle upon Tyne, UK.

[8]Institute of Molecular, Cell and Systems Biology, University of Glasgow, Glasgow G12 8QQ, UK.

[9] Departamento de Psicología Experimental, Facultad de Psicología, Universidad de Sevilla, Seville Spain.

[10] Instituto de Investigación e Innovación en Ciencias Biomédicas de Cádiz (INiBICA), Cadiz, Spain.

Running Title: Disrupted autophagy and mTOR in Tay-Sachs disease.

<u>Corresponding Authors</u>:

Dr. Mario D. Cordero
Instituto de Investigación e Innovación en Ciencias Biomédicas de Cádiz (INiBICA), Cadiz, Spain, Email: mario.cordero@inibica.es

Supplementary methods

Reagents.

Trypsin and bafilomycin A1 were purchased from Sigma Chemical Co., (St. Louis, Missouri). Anti-GAPDH monoclonal antibody from Calbiochem-Merck Chemicals Ltd. (Nottingham, UK). MnSOD, catalase and OGG-1 antibodies from Adipogen (San Diego, USA). Phospho-mTOR, mTOR, phosphor-AKT, AKT, LC3, p62, LAMP-I, Cathepsin B, HexA and galectin-3 were obtained from Cell Signaling Technology. A cocktail of protease inhibitors (complete cocktail) was purchased from Boehringer Mannheim (Indianapolis, IN). Grace's insect medium was purchased from Gibco. The Immun Star HRP substrate kit was from Bio-Rad Laboratories Inc. (Hercules, CA).

Structural mutation analysis

Human HexA and HexB cDNA sequences were downloaded from GenBank (Gene ID: 3073 and 3074) and used to interpret the functional effects of the mutations in this autosomal recessive disease with the web application Mutalyzed [1]. To investigate the effect of the mutations on protein folding, cognate 3D crystal structures were obtained from the PDB database (HEXA: 2GJX and HEXB: 1NOU) and viewed using PyMOL. The stability of the mutated structures was examined with the CUPSAT and SDM programmes; these have default values and enable torsion angles and free energy to be estimated, thus allowing changes in protein stability to be predicted.

Determination of β-hexosaminidase activities

Lysosomal hexosaminidase activities were measured as described previously [2]. Briefly, total β-hexosaminidase activity (A, B and S isozymes) was measured using the fluorogenic 4-methylumbelliferyl-2-acetamido-2-deoxy-D-glucopyranoside (4-MUG) substrate, whereas the HexA isozyme activity was determined with the sulphated, 4-

methylumbelliferyl-7-[6-sulpho-2-acetamido2-deoxy-β-D-glucopyranoside] (4-MUGS) substrate, which is preferentially hydrolysed by the A and minor S isozymes.

Western Blotting

Whole cellular lysate from fibroblasts was prepared by gentle shaking with a buffer containing 0.9% NaCl, 20 mM Tris-ClH, pH 7.6, 0.1% Triton X-100, 1 mM phenylmethylsulfonylfluoride and 0.01% leupeptin. The protein content was determined by the Bradford method. Electrophoresis was carried out in a 10–15% acrylamide SDS/PAGE and proteins were transferred to Immobilon membranes (Amersham Pharmacia, Piscataway, NJ). Next, membranes were washed with PBS, blocked over night at 4°C and incubated with the respective primary antibody solution (1:1000). Membranes were then probed with their respective secondary antibody (1:2500). Immunolabeled proteins were detected by chemiluminescence method (Immun Star HRP substrate kit, Bio-Rad Laboratories Inc., Hercules, CA). Western blot images were quantified using ImageJ software.

Electron microscopy

Fibroblasts were fixed for 15 min in the culture plates with 1.5% glutaraldehyde in culture medium and then for 30 min in 1.5% glutaraldehyde-0.1 M NaCacodylate/HCl, pH 7.4. After three washes in 0.1 M sodium cacodylate/HCl, pH 7.4 for 10 min, the cells were post-fixed and stained with 1% aqueous osmium tetroxide, pH 7.4 for 30 min. After dehydration in increasing concentrations of ethanol, 5 min for each step: 50,70, 90 and three cycles at 100% ethanol, the cells were impregnated and included in Epon, which was finally polymerised at 60°C for 48 h. 60–80 nm sections were cut in a Leica ultracut S ultramicrotome (Leitz Microsystems, Wetzlar, Germany) and contrasted with uranyl acetate and lead citrate. Cell ultrastructure was examined by electron transmission microscopy (Zeiss LEO 906 E; Oberkochen, Germany).

Proliferation rate

Two hundred thousand fibroblasts were cultured with or without the addition of L-arginine, L-leucine or DL-acetyl-leucine at different concentrations (1, 10, 5mM) for 24, 48, 72, and 120h. After discharging supernatant with dead cells, cells from three high-power fields were counted with an inverted microscope using a 40X objective

Cathepsin B and HexA release

CatB and HexA redistribution from lysosomes/autophagolysosomes to the cytosol was assessed by immunofluorescence using antibodies against CatB or HexA and LAMP-I as a marker of lysosomal/autophagolysosomal compartment. In control fibroblasts, CatB-specific immunostaining shows punctate cytoplasmic structures surrounded by lysosomal/autophagolysosomal membrane proteins such as LAMP-I. After lysosomal permeabilisation, immunofluorescence of CatB or HexA reveals diffuse staining throughout the entire cell.

Galectin 3 puncta

Lysosomal permeabilisation was also evaluated by quantification of intracellular galectin puncta, as previously described.

Protein synthesis

Briefly, the cells were incubated with serum-free DMEM for 90 min followed by incubation with 1 μM puromycin (an analogue of tyrosyl-tRNA; Invitrogen; A11138-03), for 30 min. The amount of puromycin incorporated into nascent peptides was then evaluated by Western blot using an antibody to puromycin.

L-arginine treatment in patients

Two patients with GM2 gangliosidosis (Juvenile Tay-Sachs disease 3 and Sandhoff disease 2, (see Supplementary table 1), who had not been exposed to any drug or vitamin/nutritional supplements, were supplemented with oral L-arginine (Nutricia) for 8 months (0.3g/Kg/day). After 8 months of treatment, heparinised blood samples were collected 24 hours after the last dose. No significant changes in routine clinical laboratory tests conducted on blood serum or plasma, including measures of renal and hepatic function, were observed (data not shown).

1. Wildeman M, van Ophuizen E, den Dunnen JT, Taschner PE. Improving sequence variant descriptions in mutation databases and literature using the Mutalyzer sequence variation nomenclature checker. Hum Mutat, 2008; 29: 6-13.
2. Tropak MB, Reid SP, Guiral M, Withers SG, and Mahuran D. Pharmacological enhancement of b-hexosaminidase activity in fibroblasts from adult Tay-Sachs and Sandhoff Patients. J. Biol. Chem. 2004;279:13478–13487.

Supplementary Table 1. Clinical characteristic of the patients

**Infantile 1**	
2 years old	Gender: Male
**Signs and symptoms:**<br>-Epilepsy<br>-Abnormal ocular movements<br>-Respiratory problems<br>-Macrocephaly<br>-Hypotonia severe	 -Startle with noise<br>- Hyperreflexia<br>-Psychomotor regression<br>-Low visual acuity<br>-Pyramidal syndrome
**Enzyme levels:**<br>-Isoenzymatic distribution %A: 33,5%<br>-Isoenzymatic distribution %B: 66,5%<br>-β-hexosaminidase total: 1916 nmol/h/mg prot<br>**-β-hexosaminidase A: 4 nmol/h/mg prot**<br>-Esfingomielinase: 14,9 nmol/17h/mg prot<br>-β-glucocerebrosidase: 14,6 nmol/h/mg prot	 RL: 84,0-97,0<br>RL: 3,0-16,0<br>RL:336-2868<br>RL:61-316<br>RL:8,5-26,0<br>RL:2,9-27,6
**Infantile 2**	
1 year old	Gender: Male
**Signs and symptoms:**<br>-Startle with noise<br>**-**Absent of cephalic support<br>**-**Psychomotor delays<br>-Respiratory problems<br>-Severe hypotonia	 -Abnormal ocular movements<br>-Hyperreflexia<br>-Cherry-red spot<br>-Dysphagia
**Biochemistry:**<br>-CPK, CDT, iron metabolism, folic acid, B12 vitamin, lactate, ammonium, copper, ceruloplasmina, immunoglobulins, homocysteine, 3-hydroxybutyrate, free fatty acids, long-chain fatty acids: Normal levels	
**Very low HexA enzymatic activity**	
**Infantile 3**	
4 years old	Gender: Female
**Signs and symptoms:**<br>-Macrocephaly<br>-Visual deficit<br>-Total absence of the language<br>-Hipotonia<br>-Absence of autonomous displacement	 -Absence of cephalic support<br>-Epilepsies<br>-Muscle spams<br>-Cherry-red spot<br>-Hipomielynization
**Biochemistry:**<br>-CPK, CDT, folic acid, B12 vitamin, lactate, ammonium, copper, ceruloplasmina, immunoglobulins, homocysteine, 3-hydroxybutyrate, free fatty acids, long-chain fatty acids: Normal levels	
**Very low HexA enzymatic activity**	
**Juvenile 1**	
14 years old	Gender: Male

Signs and symptoms:

-Development delays
-Startle with noise
-Facial dysmorphia
-Macrocephaly
-Slight psychomotor development delay
-Drops-attacks episodes
-Motor and language regression
-Dysphagia
-Myelination alteration
-Slow brain activity
-Visual deficit

Biochemistry:

-Low levels of HDL
-High levels of AST and LDH
-Lactate, ammonium, amino acids, iron profile, creatine, guanidinoacetate, organic acids, GAGs, 4-OH butyrate: Normal levels

Low levels of HexA activity: 3nmol/h/mg prot RL: 61-316 3nmol/h/mg prot

Juvenile 2

13 years old Gender: Female

Signs and symptoms:

-Severe dysphagia
-Motor delays
-Language regression (skills)
-Skills and motor regression
-Somatocraneal disproportion
-Slow movements
-Cherry-red spot

Biochemistry:

-CPK, ammonium, lactate, ceruloplasmin, iron profile, B12 vitamin, folic acid, amino acids, long-chain fatty acids: Normal levels

Low levels of HexA

Juvenile 3

16 years old Gender: Female

Sings and symptoms:

-Language regression
-Dysphagia
-Dysarthria
-Motor delays
-Lumbar hyperlodosis

Low HexA enzymatic activity 6% of activity

65% of discapacity

Supplementary Table 2. Genetic characteristic of the patients.

Patient	Nº Alelle	Mutation	Affects Stability?	Affects active site?
Infantile 1	Alelle 1	Premature STOP codon	Yes	Yes
	Alelle 2	Splicing	Unknown	Unknown
Infantile 2	Alelle 1	Near splicing site	Unknown	Unknown
	Alelle 2	Near splicing site	Unknown	Unknown
Infantile 3	Alelle 1	Near splicing site	Unknown	Unknown
	Alelle 2	Near splicing site	Unknown	Unknown
Juvenil 1	Alelle 1	Point	No	No
	Alelle 2	Point	Yes	Yes
Juvenil 2	Alelle 1	Point	Yes	Yes
	Alelle 2	Point	Unknown	Unknown
Juvenil 3	Alelle 1	Splicing	Unknown	Unknown
	Alelle 2	Point (Synonymous substitution)	No	No
Sandhoff 1	Alelle 1	Point	Yes	No
	Alelle 2	Intron	No	No
Sandhoff 2	Alelle 1	Premature STOP codon	Yes	Yes
	Alelle 2	Intron	No	No

Supplementary Table 3. **S**ignificant changes in mTOR pathway genes in patients *vs* control (ANOVA p-value <0.05, >1.5 fold).

Juvenile			
**Gene Symbol**	**Fold-Change**	**ANOVA p-value**	**Gene IDs**
PIK3R1	12,94	0,001497	NM_001242466
RPS6KA2	3,23	0,01184	NM_001006932
ATP6V1B2	2,84	0,035385	NM_001693
FNIP2	2,39	0,007737	NM_020840
RPS6KA1	1,69	0,044762	ENST00000627677
IRS1	1,55	0,031457	NM_005544
CLIP1	-1,49	0,01208	NM_001247997
HRAS	-1,72	0,035889	NM_005343
ATP6V1C1	-1,73	0,02705	NM_001695
RICTOR	-1,91	0,0558	NM_001285439
PRKAA2	-2,71	0,040698	NM_006252
WNT5B	-2,85	0,022171	NM_030775
RPS6KA6	-4,34	0,007977	NM_014496
RNF152	-6,08	0,009278	NM_173557
WNT5A	-47,42	0,003877	NM_001256105
**Infantile**			
**Gene Symbol**	**Fold-Change**	**ANOVA p-value**	**Gene IDs**
PIK3R1	12,95	2,06E-05	NM_001242466
LRP5	3,81	0,0045	NM_001291902
RPS6KA2	2,51	0,0337	NM_001006932
FNIP2	2,12	0,0207	NM_020840
LRP6	2,04	0,0392	ENST00000628182
ATP6V1E2	2,03	0,043	NM_080653
WNT11	2	0,0283	NM_004626
ATP6V1B2	1,93	0,0207	NM_001693
TNFRSF1A	1,88	0,0197	NM_001065
EIF4E2	1,86	0,0489	NM_001276336
FZD1	1,84	0,0387	NM_003505
NPRL3	1,79	0,0382	NM_001039476
ATP6V1G3	1,54	0,0105	NM_133262
FZD5	-1,48	0,0182	NM_003468
SLC38A9	-1,55	0,0502	NM_001258286
RAF1	-1,78	0,0051	NM_002880
ATP6V1C1	-1,83	0,0037	NM_001695
AKT1	-1,89	0,0477	NM_001014431
WNT2B	-1,95	0,0126	NM_001291880
PIK3R2; IFI30	-2,01	0,0325	NM_005027
PRKAA2	-2,03	0,0205	NM_006252

SEH1L	-2,33	0,0121	NM_001013437
IGF2; INS-IGF2	-2,46	0,0003	NM_001007139
WNT5B	-2,74	0,015	NM_030775
RPS6KA6	-2,8	0,0027	NM_014496
RICTOR	-2,83	0,0018	NM_001285439
LPIN1; MIR548S	-4,22	0,0083	NM_001261427
RNF152	-8,06	6,21E-06	NM_173557
**Sandhoff**			
**Gene Symbol**	**Fold-Change**	**ANOVA p-value**	**Gene IDs**
PIK3R1	10,84	0,038841	NM_001242466
FNIP2	3,05	0,013828	NM_020840
STRADA	2,31	0,028354	NM_001003786
LRP6	2,02	0,00027	NM_002336
IGF1R	1,95	0,034823	NM_000875
ATP6V1C1	-1,64	0,044767	NM_001695
RAF1	-1,74	0,036988	NM_002880
PRKCA	-2,95	0,020134	NM_002737
RPS6KA6	-3	0,017731	NM_014496
WNT5A	-3,91	0,026136	NM_001256105
RNF152	-10,18	0,007434	NM_173557

Supplementary Table 4. Significant changes in Autophagy pathway genes in patients *vs* control (ANOVA p-value <0.05, >1.5 fold).

Infantile			
**Gene Symbol**	**Fold-Change**	**ANOVA p-value**	**Gene IDs**
PIK3R1	12,95	2,06E-05	NM_001242466
RAB7B	5,1	0,0001	NM_001164522
RRAS2	3,31	0,0022	NM_001102669
DAPK1	2,26	0,0281	NM_001288729
ZFYVE1	2,05	0,0204	NM_001281734
HIF1A	2,03	0,0277	NM_001243084
GABARAP	1,87	0,0073	NM_007278
BAD	1,84	0,0049	NM_004322
CTSB	1,72	0,034	NM_001908
ATG10	1,64	0,0259	NM_001131028
RAF1	-1,78	0,0051	NM_002880
AKT1	-1,89	0,0477	NM_001014431
PIK3R2; IFI30	-2,01	0,0325	NM_005027
PRKAA2	-2,03	0,0205	NM_006252
ATG4D	-2,17	0,0145	NM_001281504
IGF2; INS-IGF2	-2,46	0,0003	NM_001007139
ATG7	-2,7	0,0022	NM_001136031
ERN1	-3,29	0,0032	NM_001433
PRKCD	-3,64	0,0003	NM_006254
**Juvenile**			
**Gene Symbol**	**Fold-Change**	**ANOVA p-value**	**Gene IDs**
PIK3R1	12,94	0,001497	NM_001242466
RAB7B	2,87	0,01827	NM_001164522
GABARAP	1,92	0,031715	NM_007278
HIF1A	1,72	0,01766	NM_001243084
ZFYVE1	1,71	0,04872	NM_001281734
IRS1	1,55	0,031457	NM_005544
HRAS	-1,72	0,035889	NM_005343
PRKCD	-2,28	0,024094	NM_006254
PRKAA2	-2,71	0,040698	NM_006252

Sandhoff			
**Gene Symbol**	**Fold-Change**	**ANOVA p-value**	**Gene IDs**
PIK3R1	10,84	0,038841	NM_001242466
RAB7B	3,68	0,042686	NM_001164522
IGF1R	1,95	0,034823	NM_000875
DAPK2	1,92	0,019391	NM_014326
PRKACG	1,83	0,0003	NM_002732
PIK3R4	1,63	0,037854	NM_014602
PIK3CD	-1,38	0,00897	NM_005026
VAMP8	-1,43	0,042707	NM_003761
PIK3C3	-1,62	0,039853	NM_001308020
RAF1	-1,74	0,036988	NM_002880
EIF2AK4	-1,88	0,016362	NM_001013703
BCL2L1	-3,26	0,011985	NM_001191
PRKCD	-3,6	0,005909	NM_006254

Supplementary Table 5. Significant changes in lysosome pathway genes in patients *vs* control (ANOVA p-value <0.05, >1.5 fold).

Juvenile			
**Gene Symbol**	**Fold-Change**	**ANOVA p-value**	**Gene IDs**
CD68	4,16	0,040966	NM_001040059
CTSO	2,68	0,037234	NM_001334
LAPTM4A	1,8	0,020544	hsa_circ_0000981
IGF2R	-1,58	0,022001	NM_000876
LITAF	-1,7	0,021841	NM_001136472
SCARB2	-1,88	0,009984	NM_001204255
NAGA	-2,18	0,021429	NM_000262
CLTCL1	-2,37	0,037322	NM_001835
NEU1	-2,57	0,048515	ENST00000375631
CTSZ	-2,82	0,030783	NM_001336
AGA	-3,03	0,044367	NM_000027
ARSG	-4,12	0,037029	NM_001267727
NPC1	-5,83	0,031279	NM_000271
SORT1	-5,89	0,010414	NM_001205228
**Infatile**			
**Gene Symbol**	**Fold-Change**	**ANOVA p-value**	**Gene IDs**
CD68	5,7	0,036	NM_001040059
CTSO	2	0,0252	NM_001334
ARSA	1,9	0,0356	NM_000487
CTSB	1,72	0,034	NM_001908
LGMN	1,62	0,0414	NM_001008530
CTSF	1,49	0,0346	NM_003793
LAPTM4B	-1,87	0,0051	NM_018407
LIPA	-2,19	0,0152	NM_000235
NEU1	-2,75	0,0067	ENST00000375631
CTNS	-2,81	0,0057	NM_001031681
AGA	-3,45	0,0006	NM_000027
ARSG	-3,52	0,0172	NM_001267727
NPC1	-7,34	0,0004	NM_000271
HEXA	-27,12	0,0075	NM_000520
**Sandhoff**			
**Gene Symbol**	**Fold-Change**	**ANOVA p-value**	**Gene IDs**
CD68	7,3	0,003732	NM_001040059
LAPTM4A	2,51	0,044028	hsa_circ_0000981
AP1G2	1,2	0,026778	NM_001282474
CLTC	-1,33	0,034873	NM_001288653
CLTA	-1,35	0,000585	NM_001076677
LITAF	-2,34	0,006126	NM_001136472
IGF2R	-2,36	0,027342	NM_000876

AGA	-3,51	0,008182	NM_000027

Supplementary Table 6. Significant changes in arginine biosynthesis pathway genes in patients *vs* control (ANOVA p-value <0.05, >1.5 fold).

**Juvenile**			
**Gene Symbol**	**Fold-Change**	**ANOVA p-value**	**Gene IDs**
CPS1	1,96	0,040666	NM_001122633
ABHD14A-ACY1	1,69	0,003892	OTTHUMT00000349691
GOT2	-3,89	0,032579	NM_001286220
GLUL	-4,21	0,003389	NM_001033044
ASS1	-12,13	0,002704	NM_000050
**Infantile**			
**Gene Symbol**	**Fold-Change**	**ANOVA p-value**	**Gene IDs**
GLS	3,03	0,018	NM_001256310
GOT1	-1,5	0,0427	NM_002079
NOS3	-1,71	0,0237	NOS3.kAug10-unspliced
GOT2	-3,71	0,0113	NM_001286220
GLUL	-5,81	6,62E-06	NM_001033044
ASS1	-8,45	0,0003	NM_000050
**Sandhoff**			
**Gene Symbol**	**Fold-Change**	**ANOVA p-value**	**Gene IDs**
ABHD14A-ACY1	2	0,005936	OTTHUMT00000349691
GOT2	-4,04	0,040265	NM_001286220
GLUL	-4,56	0,009998	NM_001033044
ASS1	-44,73	0,013876	NM_000050

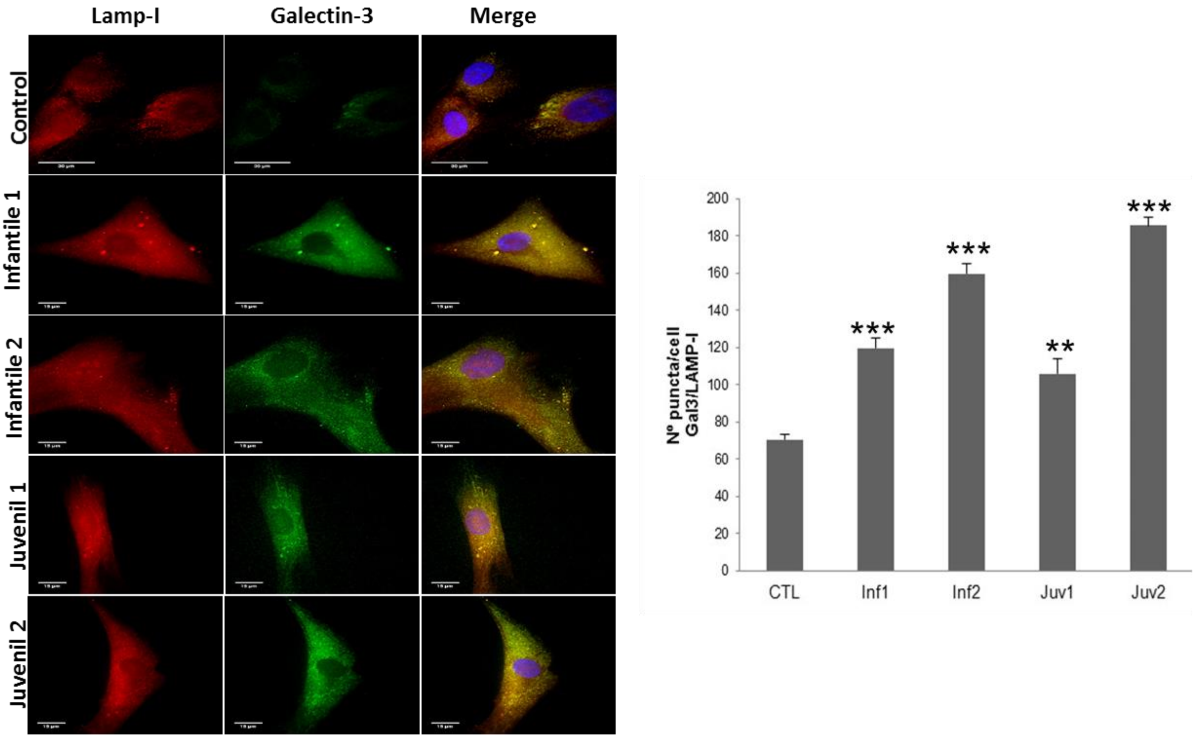

Supplementary Figure 1. Representative fluorescence images and quantification of fibroblasts from control and TSD. Cells were fixed and stained with anti-Galectin-3 antibodies (green) and anti-LAMP-I (red). Nuclei were stained with Hoechst 33342 (blue). Increased Galectin-3- puncta and colocalization of Galectin-3 and LAMP-I puncta are shown in patients. The data are the mean ± SD for experiments conducted on 2 different control cell lines and three separate experiments. ***$p < 0.001$, **$p < 0.005$, *$p < 0.05$ between controls and TSD patients.

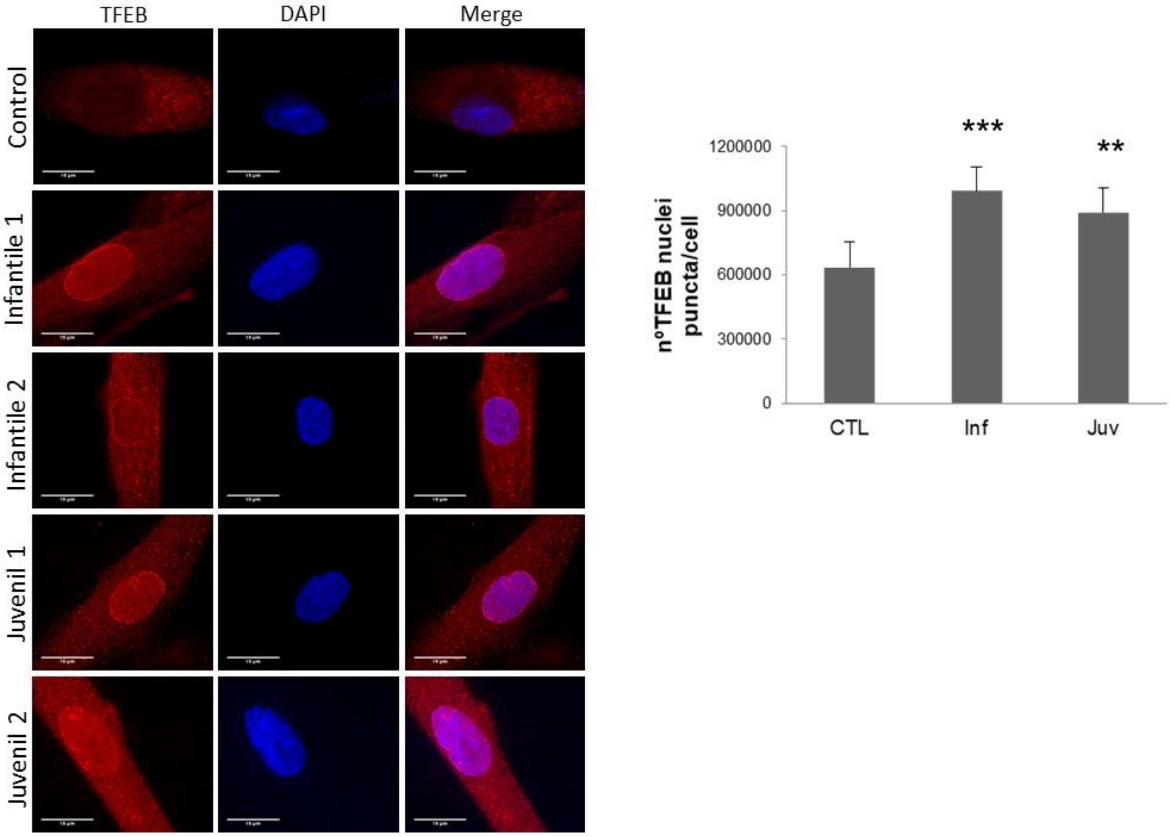

Supplementary Figure 2. Representative fluorescence images and quantification of fibroblasts from control and Tay-Sachs (TSD). Cells were fixed and stained with anti-TFEB antibodies (red). Nuclei were stained with Hoechst 33342 (blue). Increased TFEB in nucleus are shown in patients by red and blue fluorescence signal colocalization.

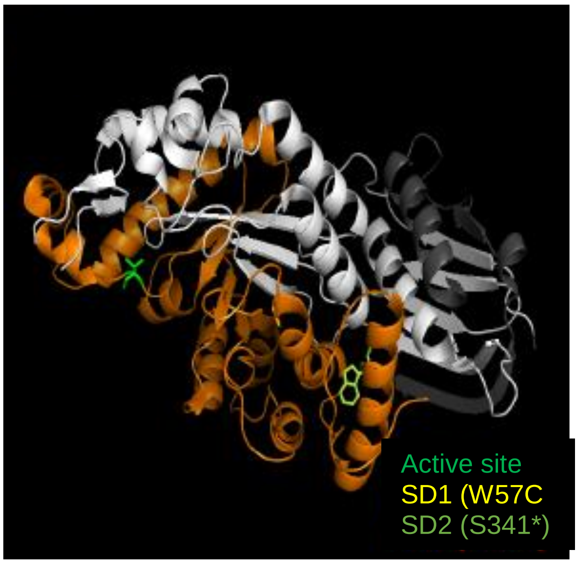

Supplementary Figure 3. HEXB point mutations and frameshifts. The active site is also highlighted. Grey colour represents the propeptide, and white colour represents the main chain of the protein. Orange color represents the sequence lost by the SD1 patient due to a frameshift.

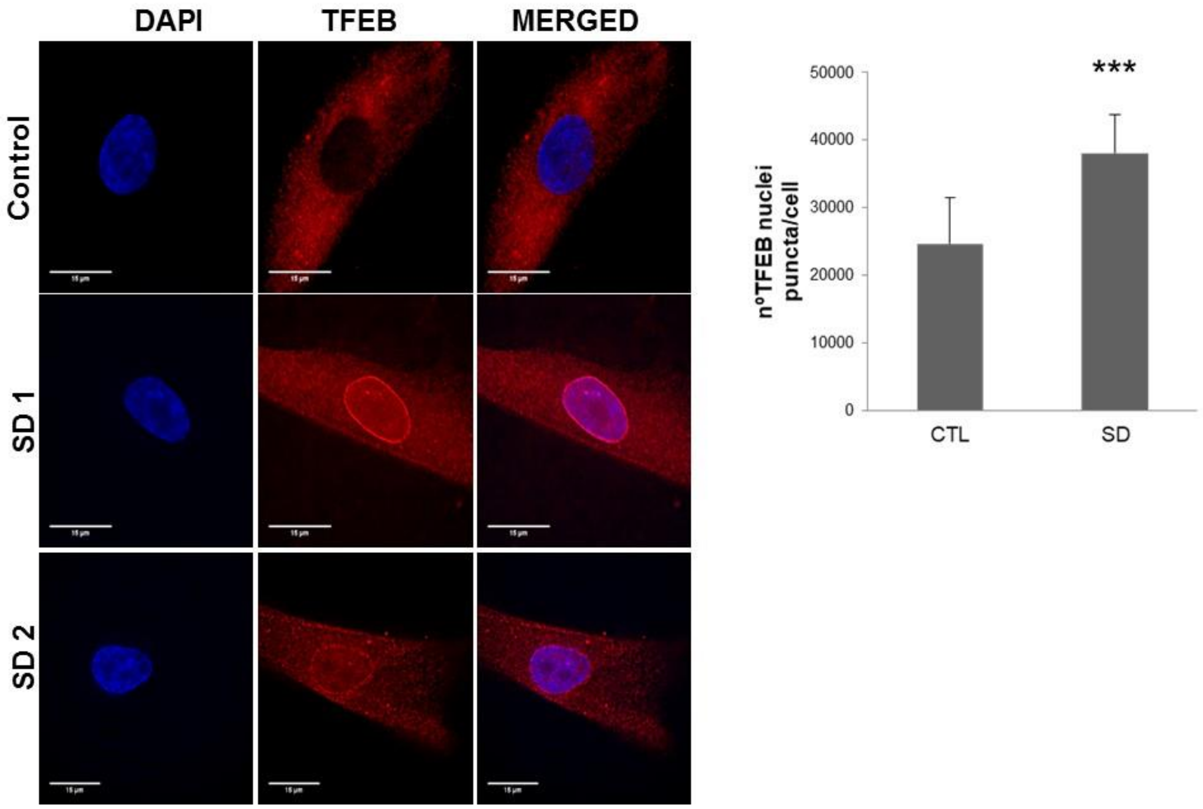

Supplementary Figure 4. Representative fluorescence images and quantification of fibroblasts from control and Sandhoff (SD). Cells were fixed and stained with anti-TFEB antibodies (red). Nuclei were stained with Hoechst 33342 (blue). Increased TFEB in nucleus are shown in patients by red and blue fluorescence signal colocalization.

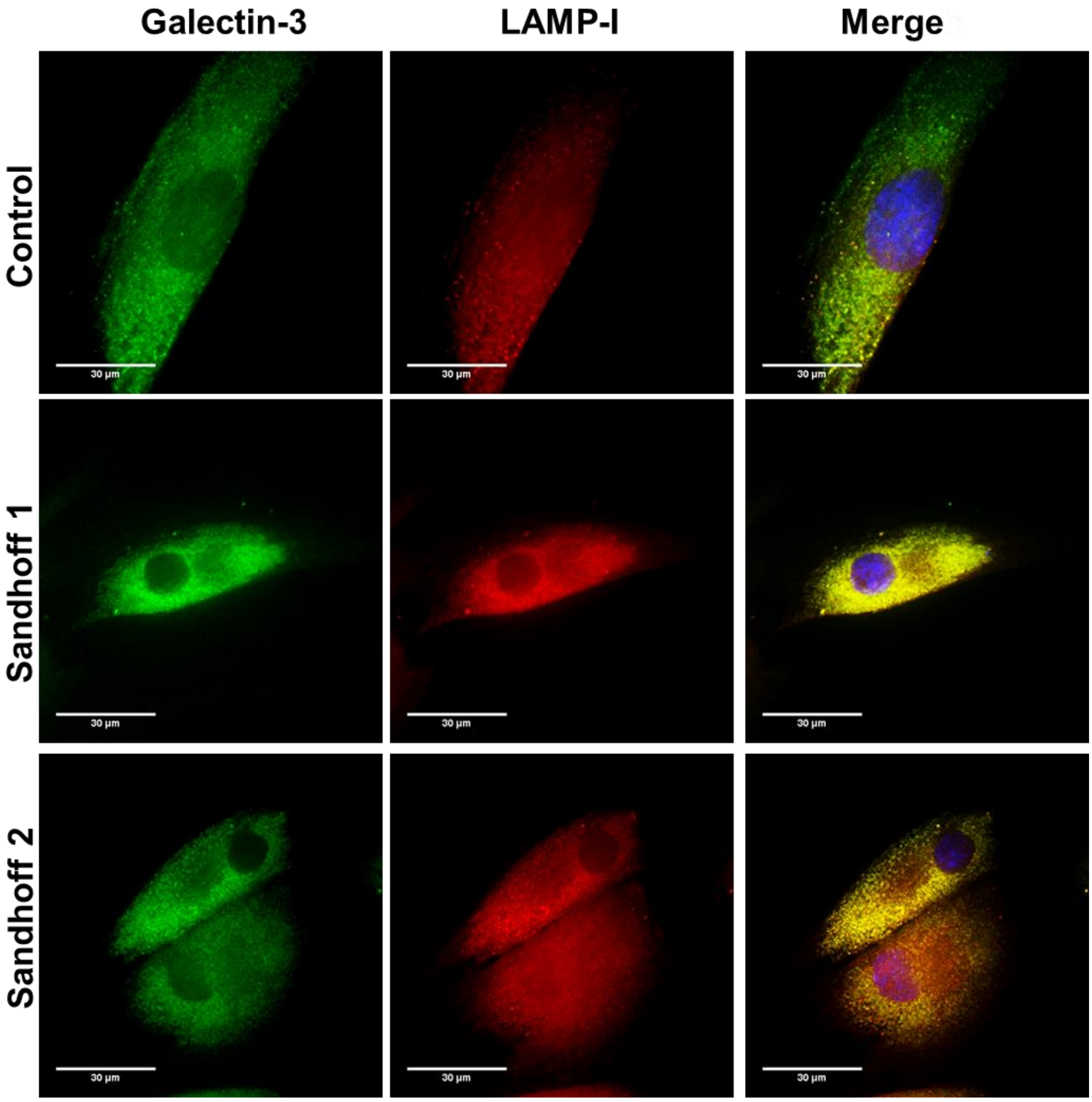

Supplementary Figure 5. Representative fluorescence images and quantification of fibroblasts from control and Sandhoff patients. Cells were fixed and stained with anti-Galectin-3 antibodies (green) and anti-LAMP-I (red). Nuclei were stained with Hoechst 33342 (blue).

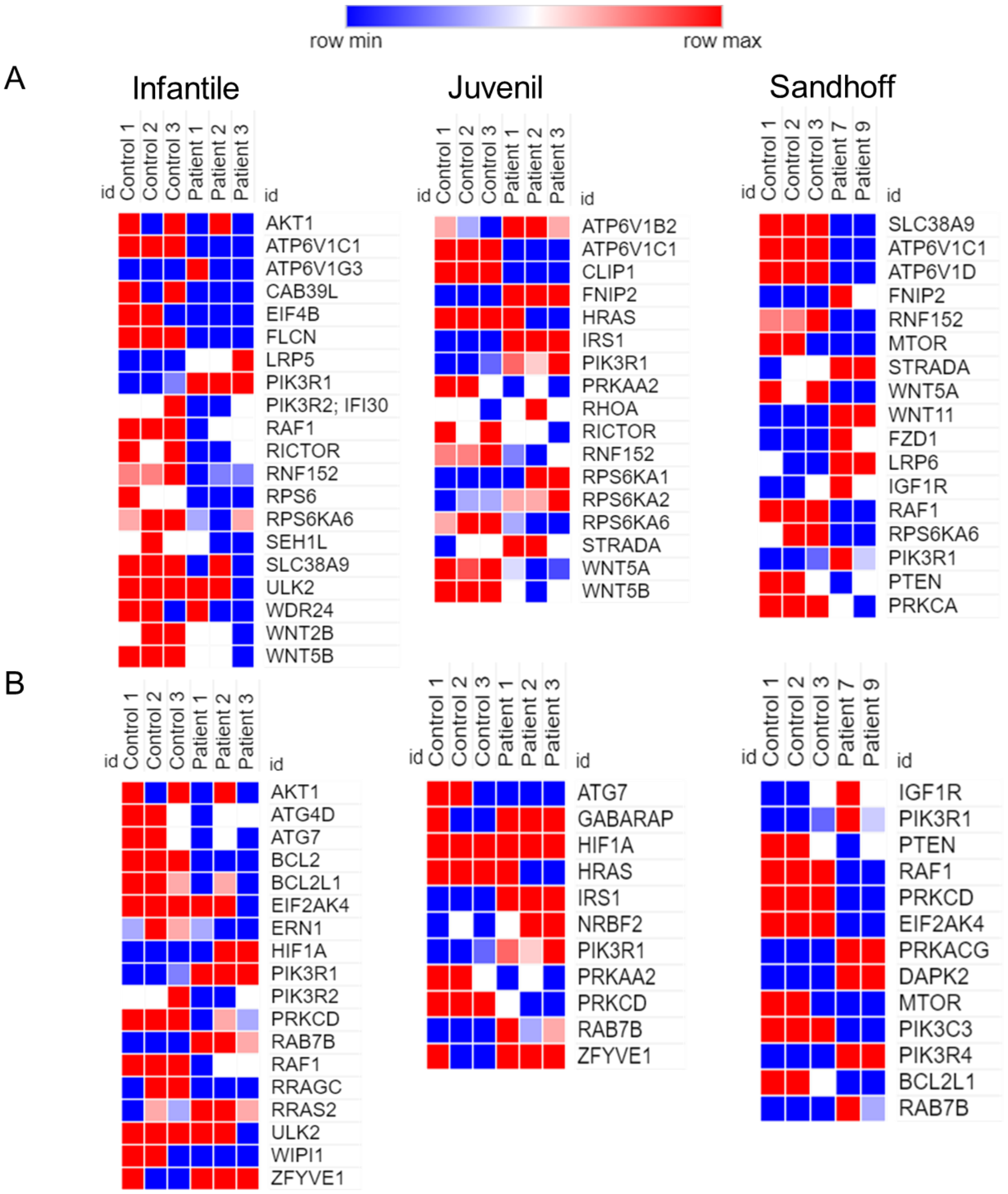

Supplementary Figure 6. Heatmap clustering of enrichment (z-scores) of the mTOR functions (A) and autophagy (B) in set of coding genes differentially expressed between control and diseased fibroblasts (n = 3 per case).

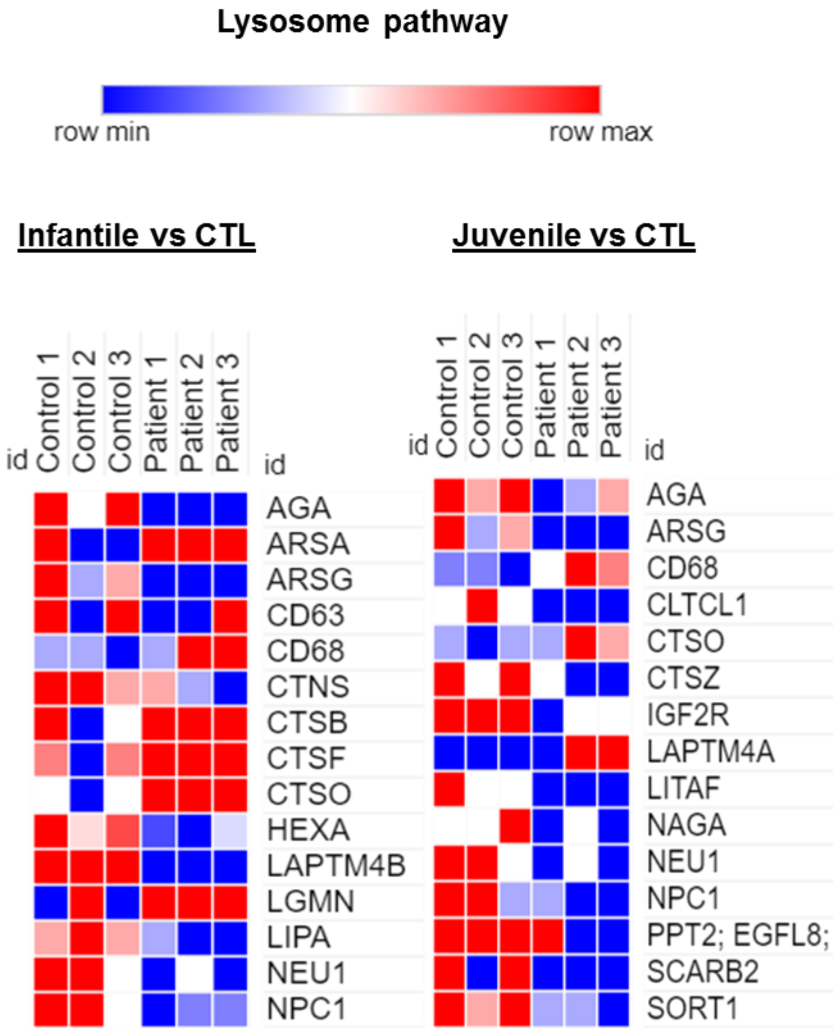

Supplementary Figure 7. Heatmap clustering of enrichment (z-scores) of the lysosome function in set of coding genes differentially expressed between Control vs Tay-Sachs patients (n = 3 per case).

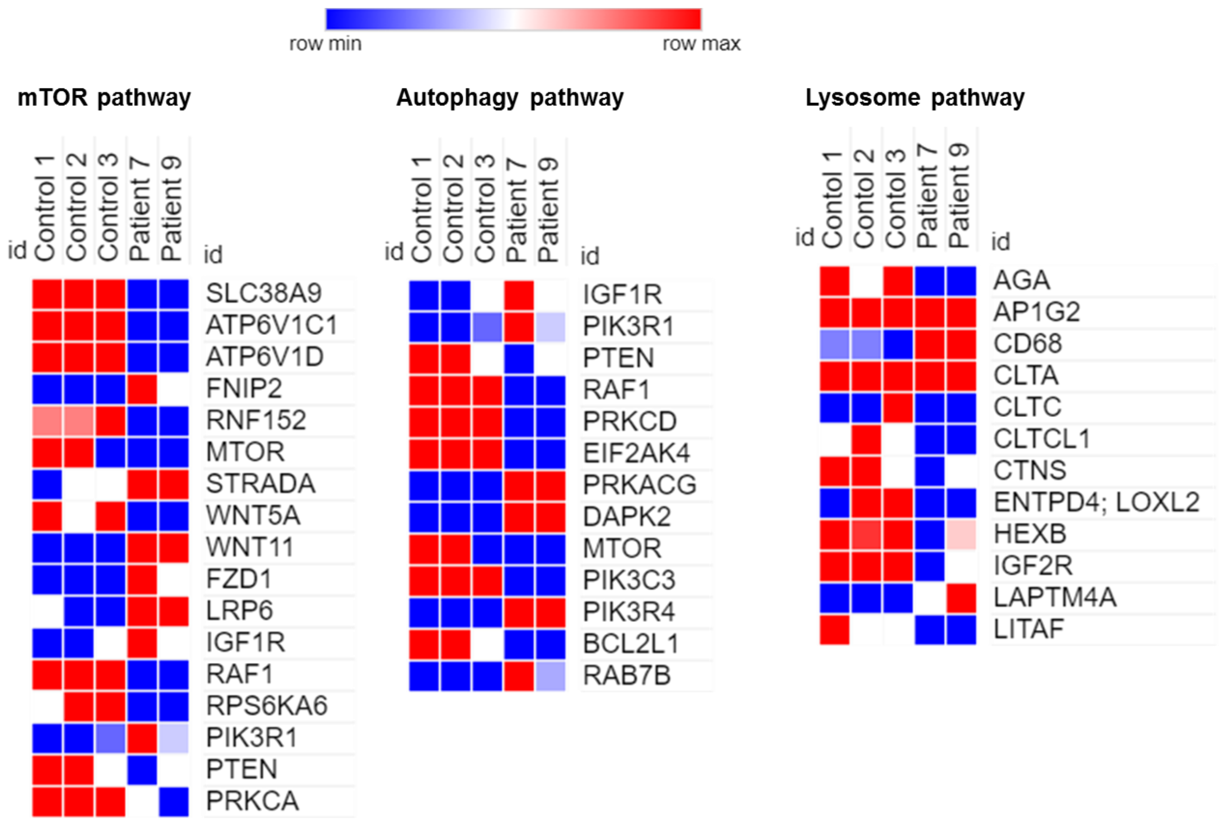

Supplementary Figure 8. Heatmap clustering of enrichment (z-scores) of the mTOR (left), autophagy (center) and lysosome (right) functions in set of coding genes differentially expressed between Control vs Sandhoff patients (n = 3 per case).

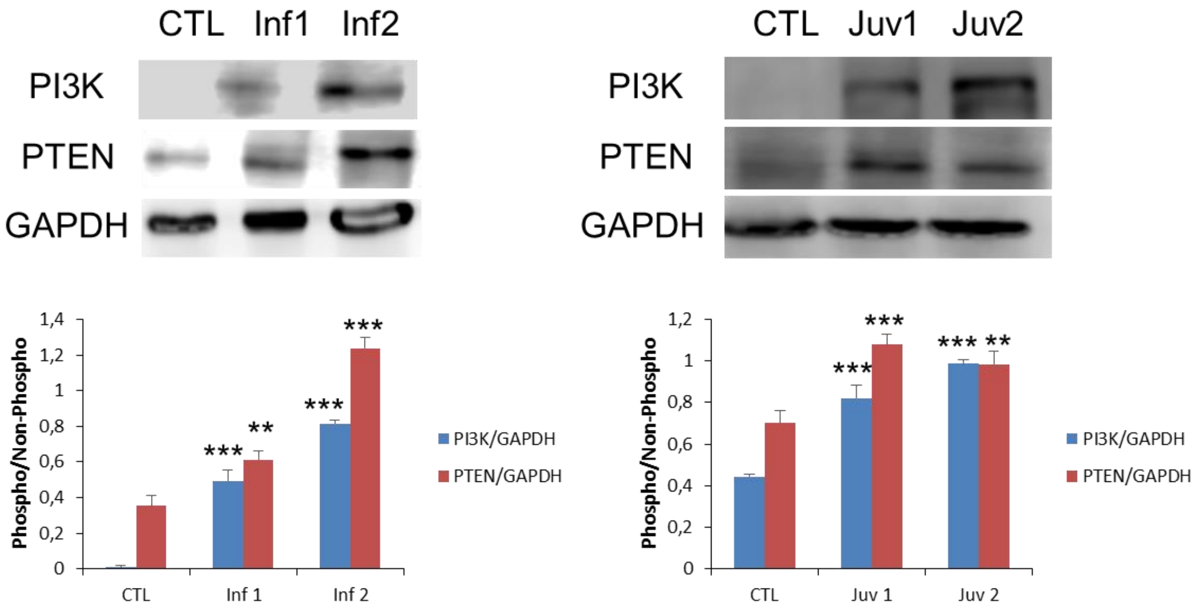

Supplementary Figure 9. Protein expression levels of PI3K and PTEN of representative control and Tay-Sachs patient fibroblasts. The data are the mean ± SD for experiments conducted on 2 different control cell lines and three separate experiments. *P < 0.05; **P < 0.01; ***P < 0.001 between control and patients.

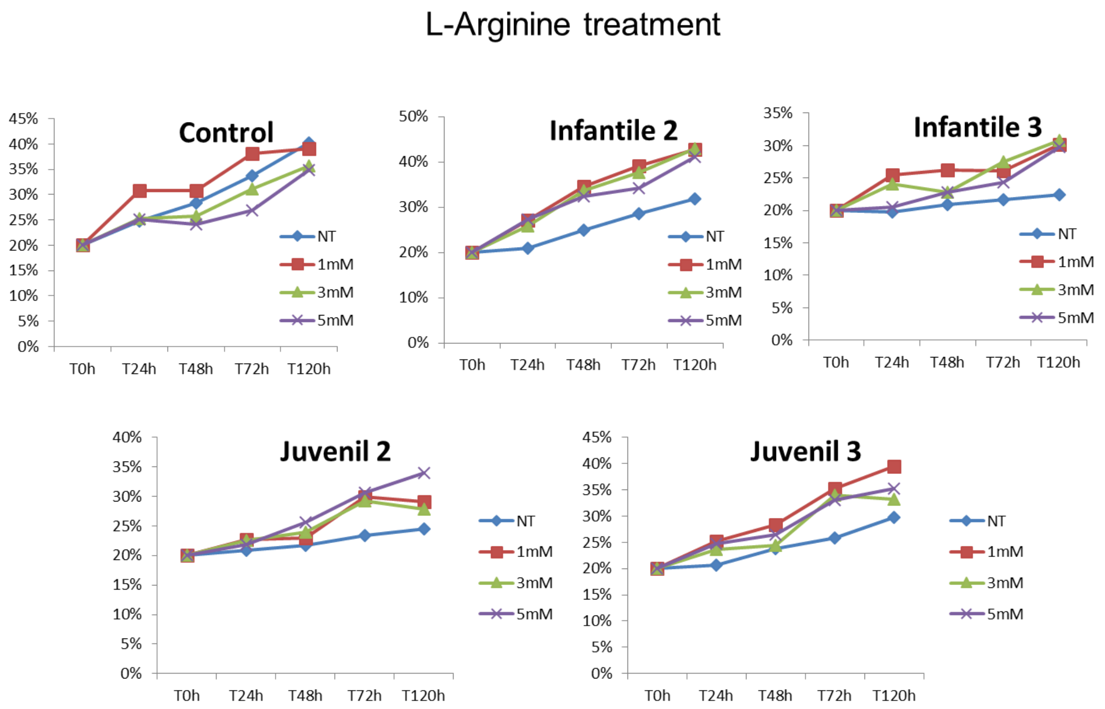

Supplementary Figure 10. Percentage of cell growth with L-Arginine determined in healthy and representative Tay-Sachs fibroblasts using three different doses (1, 3 and 5mM).

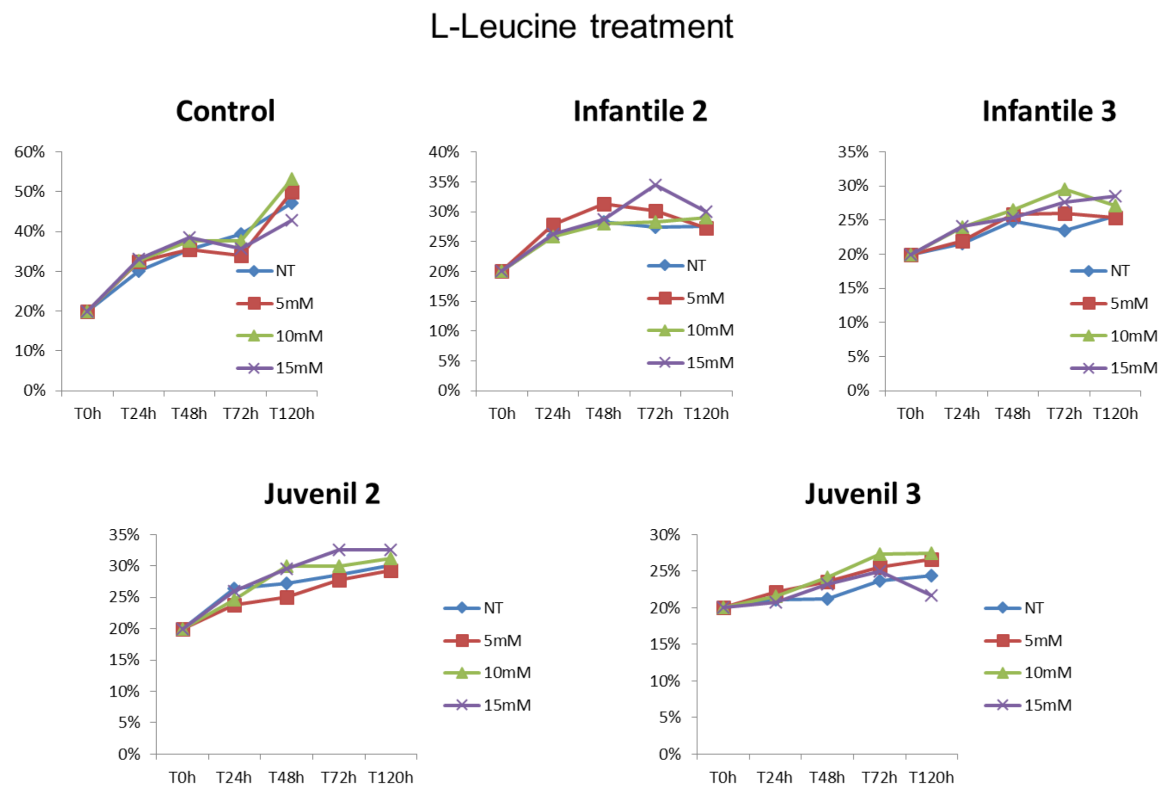

Supplementary Figure 11. Percentage of cell growth with L-Leucine determined in healthy and representative Tay-Sachs fibroblasts using three different doses (5, 10 and 15mM).

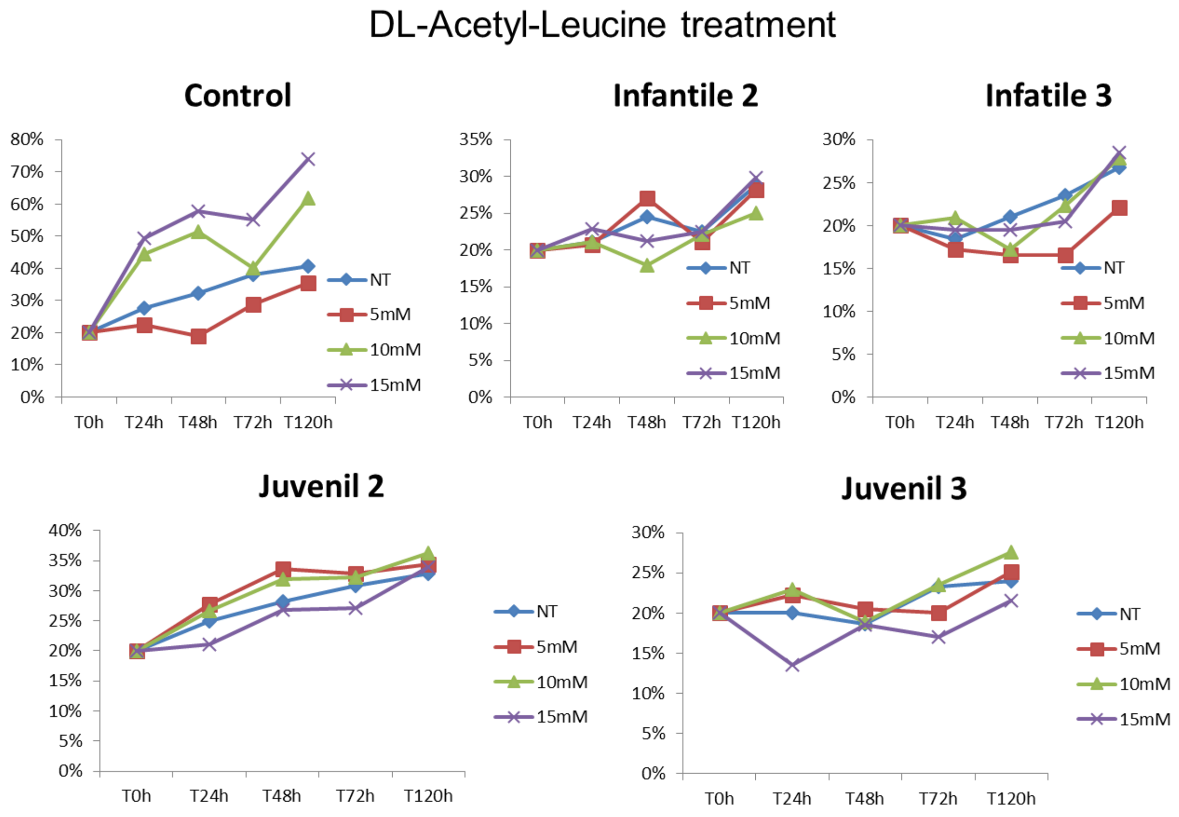

Supplementary Figure 12. Percentage of cell growth with DL-Acetyl-Leucine determined in healthy and representative Tay-Sachs fibroblasts using three different doses (5, 10 and 15mM).

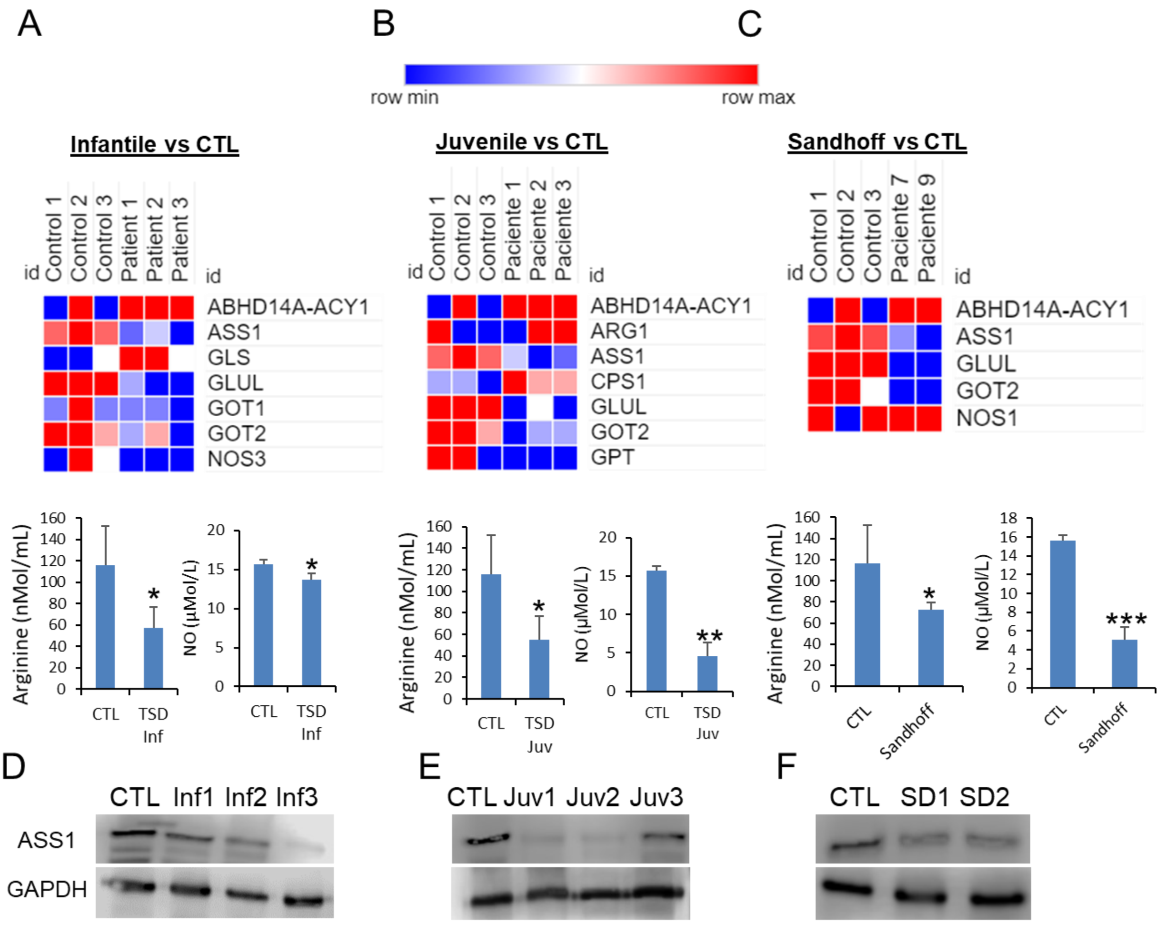

Supplementary Figure 14

Supplementary Figure 13. Heatmap clustering of enrichment (z-scores) of the arginine biosynthesis functions in set of coding genes differentially expressed and serum arginine and nitric oxide levels in infantile Tay-Sachs (TSD) (A), juvenil TSD (B) and Sandhoff (SD) (C) (n = 3 per case). Protein expression levels of ASS1 in fibroblasts from infantile TSD (D), juvenil TSD (E) and SD (F). The data are the mean ± SD for experiments conducted on 2 different control cell lines and three separate experiments. *P < 0.05; **P < 0.01; ***P < 0.001 between control and patients;

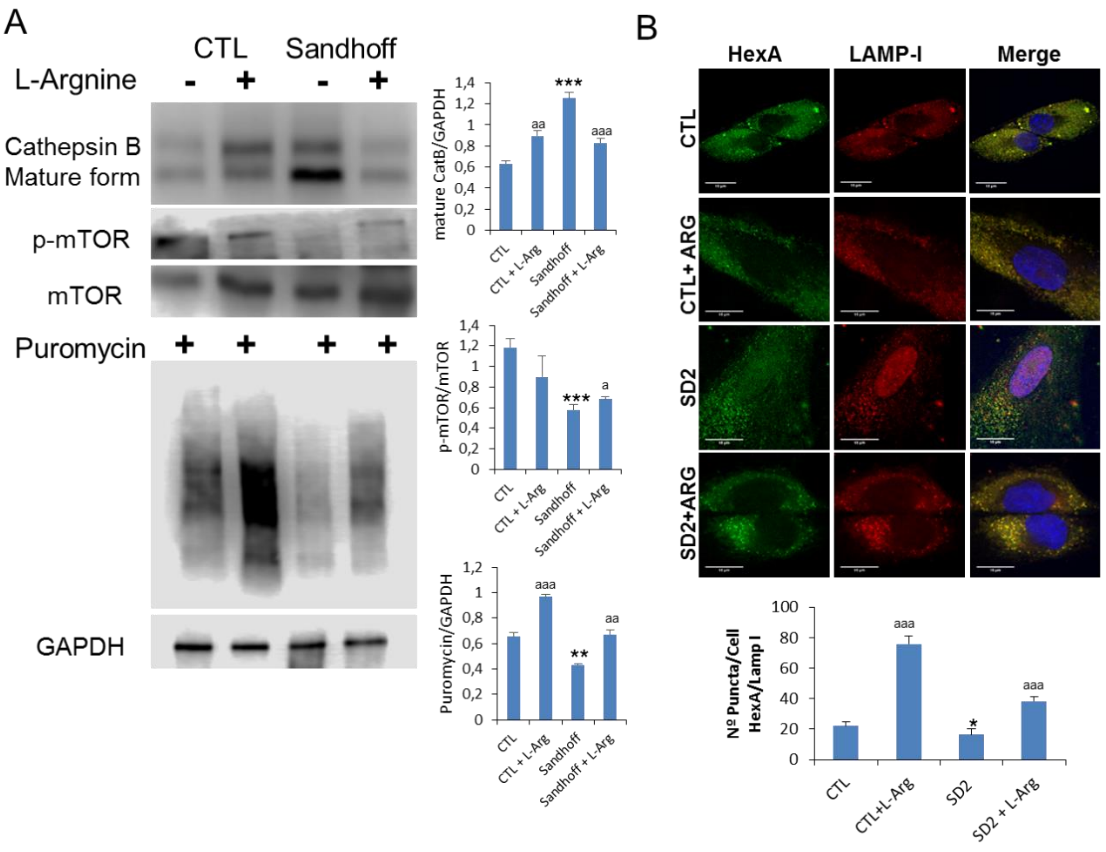

Supplementary Figure 14. A. Protein expression levels of mTOR and CatB were determined in control and representative SD fibroblast cultures after l-arginine treatment (120h). Protein synthesis was quantified in protein extracts of control and Sandhoff (SD) fibroblasts treated with l-arginine using puromycin labeling followed by immunoblot. B. Immunofluorescence of HexA in control and SD cells and quantification after l-arginine treatment. The data are the mean ± SD for experiments conducted on 2 different control cell lines and three separate experiments. *P < 0.05; **P < 0.01; ***P < 0.001 between control and SD patients; [a] P < 0.05; [aa] P < 0.01; [aaa] P < 0.001 between non-treated and treated cells.

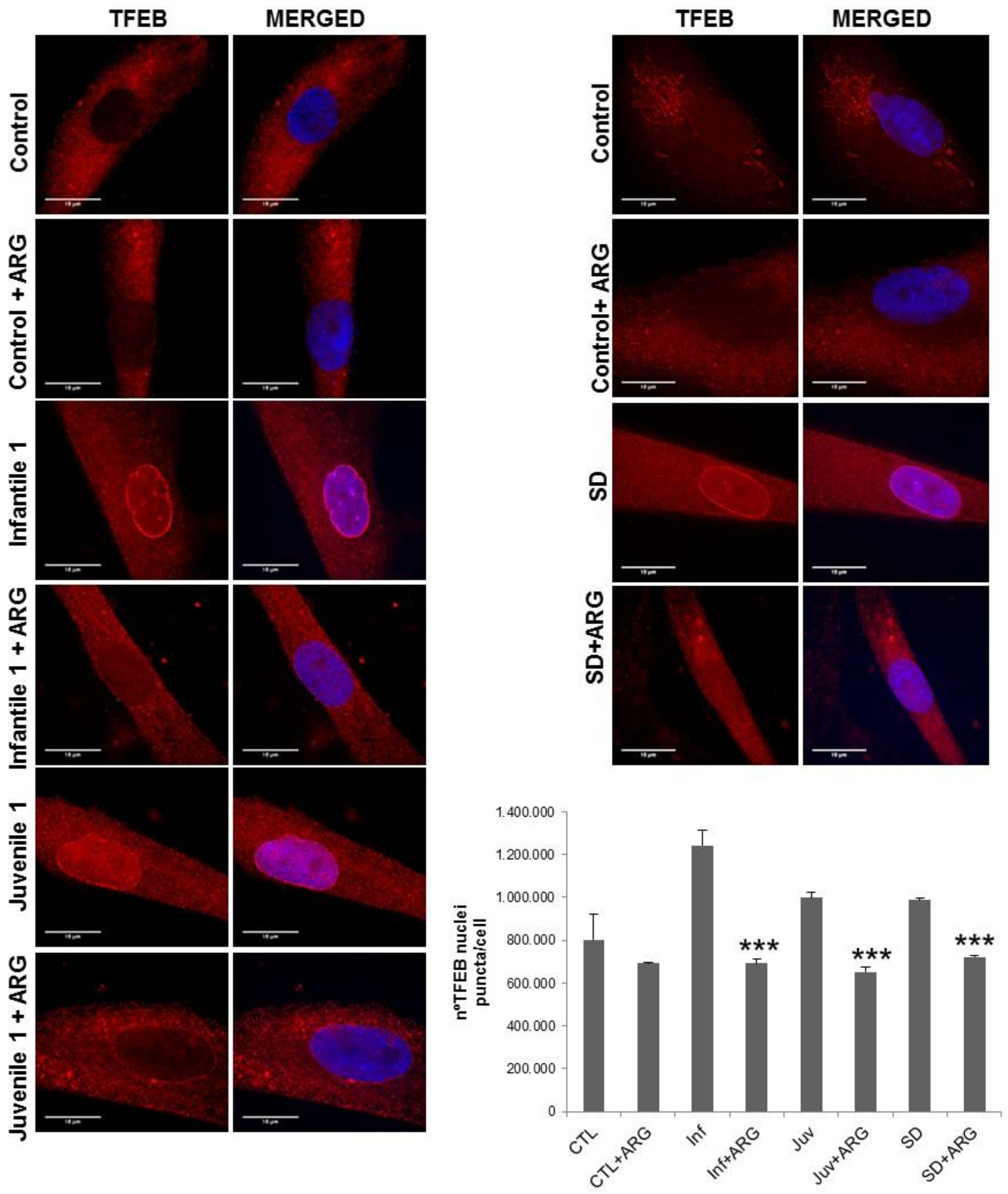

Supplementary Figure 15. Representative fluorescence images and quantification of fibroblasts from control, Tay-Sachs (infantil and juvenile) and Sandhoff (SD) with and without L-arginine treatment. Cells were fixed and stained with anti-TFEB antibodies (red). Nuclei were stained with Hoechst 33342 (blue). Increased TFEB in nucleus are shown in patients by red and blue fluorescence signal colocalization which was reduced after L-arginine treatment.
